# Post-traumatic Secondary Complications Resembling Stroke Symptoms Following Axillofemoral Bypass Surgery: A Case Report

**DOI:** 10.7759/cureus.65660

**Published:** 2024-07-29

**Authors:** Daniel Levine, Allen Zhang, Khalid Haikal, Paul Janda, Aroucha Vickers

**Affiliations:** 1 Medical School, Kirk Kerkorian School of Medicine at UNLV (University of Nevada, Las Vegas), Las Vegas, USA; 2 Neurology, Valley Hospital Medical Center, Las Vegas, USA

**Keywords:** pad (peripheral artery disease), subclavian arterial injury, brachial plexus injury, stroke mimics, axillofemoral bypass graft

## Abstract

Peripheral artery disease exerts a substantial toll on public health in the United States, straining healthcare resources. In challenging cases, the axillofemoral bypass graft had emerged as a cornerstone in managing this condition. Unforeseen events, such as trauma, can lead to a presentation mimicking stroke and thus exacerbating the complexity of the diagnostic process. Herein, we present the case of a 64-year-old male with complex peripheral artery disease who developed a pseudoaneurysm mimicking stroke symptoms following a traumatic incident post axillofemoral bypass graft surgery. This case underscores the critical importance of employing diverse diagnostic modalities to navigate the complex differential diagnosis of stroke-like symptoms in patients post-surgery.

## Introduction

Arterial constriction, or peripheral artery disease (PAD), affects nearly nine million individuals in the United States alone. It arises from the accumulation of plaque within the vessel's intima. This plaque buildup leads to obstructions that, when severe, restrict blood flow to affected areas, potentially causing ischemia and infarction. The primary goal in treating PAD is to alleviate symptoms, preserve function, and mitigate cardiovascular risk. Management typically involves lifestyle adjustments, and if these prove ineffective, revascularization may be necessary [1]. Balloon angioplasty, often combined with stenting, is commonly the first choice to widen the vessel lumen and enhance blood flow [2]. In cases where endovascular methods are unfeasible, open surgical intervention, such as the axillofemoral bypass graft (AXFBG), becomes necessary to restore blood flow to the limbs [1].

First introduced in 1963, the AXFBG became the "gold standard" surgical technique for treating severe occlusive disease [1,3,4]. However, over time, it has not been used as frequently with the main use in critical situations such as addressing limb ischemia resulting from aortic thrombosis dissection or graft infections [5-9]. It is recommended for patients with challenging abdominal conditions, a history of multiple prior abdominal surgeries, elevated risks with general anesthesia, or those who are critically unwell [3,10,11]. The procedure establishes a connection between the axillary and the common femoral arteries [1].

Injuries to the subclavian vessels following this procedure carry a high level of risk, given their potential to inflict severe neurological damage due to their proximity to the brachial plexus. The injury typically arises from either penetrating or blunt trauma to the artery. Early identification and intervention to address the underlying cause of the injury are of paramount importance for effective functional recovery [4]. While instances of pseudoaneurysms resulting from this procedure are rare, there have been some documented cases [12,13]. What distinguishes our case is the relationship to trauma following the procedure that impacts the adjacent structures, leading to compression of the brachial plexus.

## Case presentation

**Figure 1:**
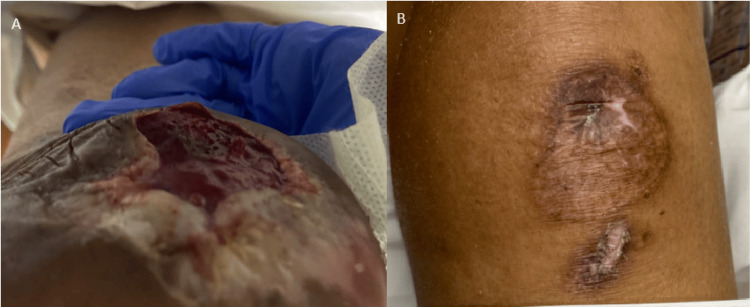
Photographs of the non-healing ulcers of the left heel (A) and left knee (B), which were the initial reason behind the patient’s hospital visit and admission

We report a case of a vasculopathic 64-year-old male who initially presented due to a non-healing wound of his lower extremities bilaterally (Figure 1). His past medical history included hypertension, hyperlipidemia, diabetes mellitus, severe peripheral artery disease, right subclavian artery stenosis status post bypass graft, and bilateral calcaneal osteomyelitis.

**Figure 2:**
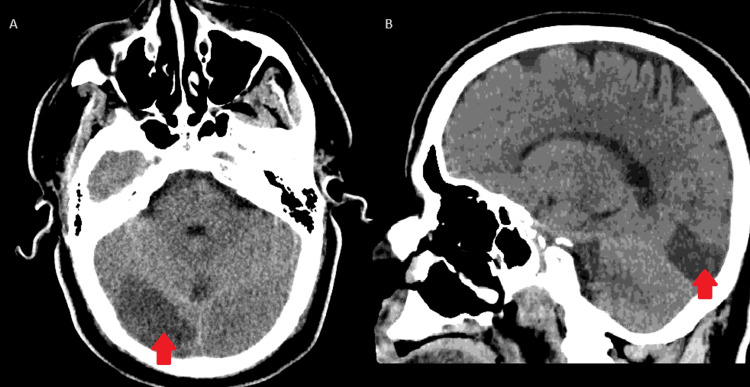
CT head with axial (A) and sagittal (B) views showing no evidence of hemorrhage but notable for a hypodensity in the right occipital lobe (red arrow), indicating the possibility of a developing ischemic infarct in the region

**Figure 3:**
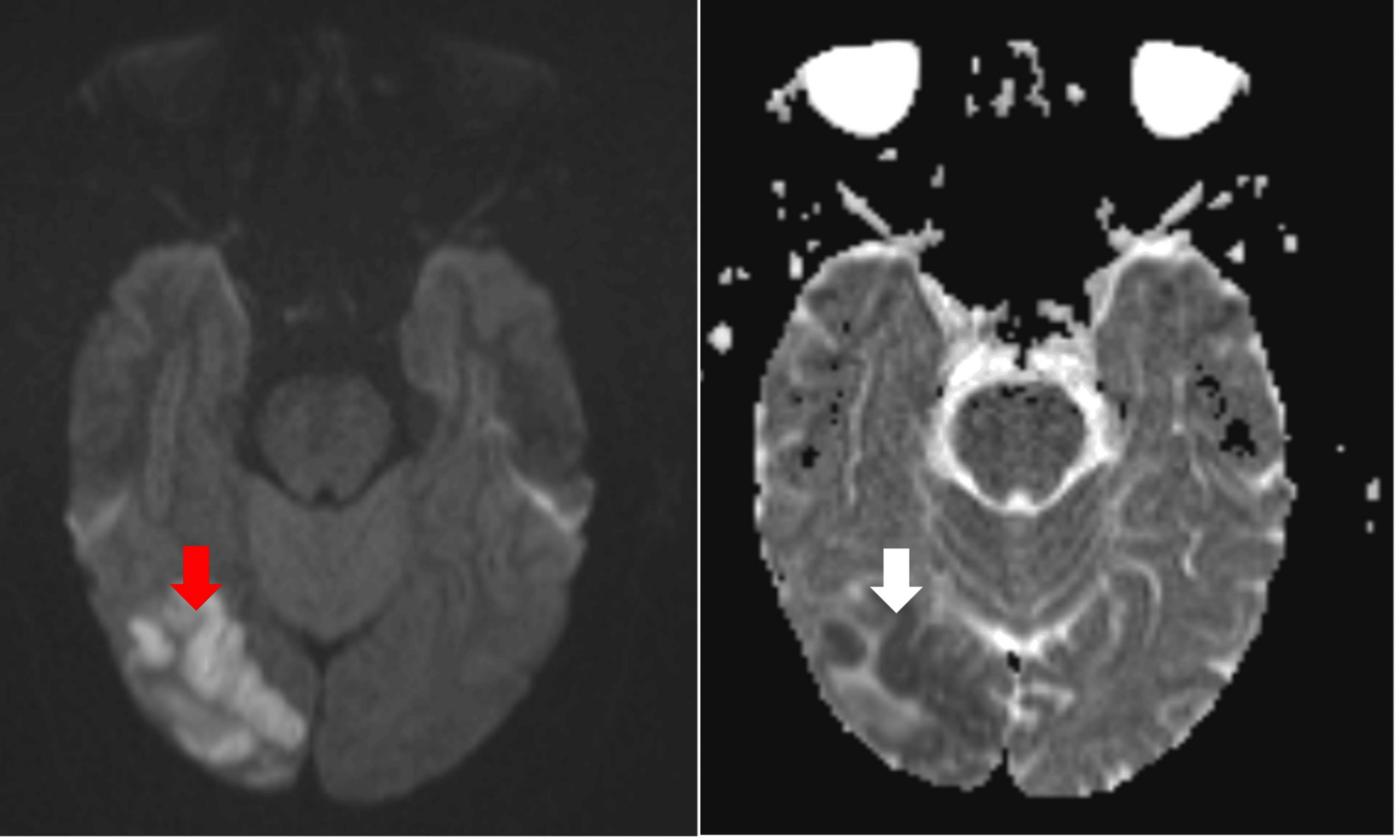
MRI brain showing restricted diffusion in the right occipital lobe on diffusion-weighted imaging (DWI) (red arrow) with corresponding hypointensity on apparent diffusion coefficient (ADC) (white arrow)

Computerized tomography (CT) of the aorta demonstrated occlusion of the infrarenal aorta with distal reconstitution of the bilateral common femoral arteries with severe superficial femoral artery disease distally. He underwent bilateral common femoral artery endarterectomy and right axillary artery to bilateral common femoral artery bypass graft. Intraoperatively, acute thrombosis of the graft occurred, requiring multiple thrombectomies and switching from heparin drip to argatroban due to concern for heparin-induced thrombocytopenia. A hypercoagulable disorder was suspected but a comprehensive hypercoagulable workup was unrevealing. Postoperatively, he was noted to have hemorrhagic anemia for which he received two units of packed red blood cells with improvement. On postoperative day one, the patient complained of a headache without new neurologic deficits. A CT of the head without contrast (Figure 2) was negative for intracranial hemorrhage but raised the possibility of a right occipital ischemic stroke. CT angiogram of the head and neck showed severe diffuse intracranial atherosclerotic disease with no significant stenosis of the proximal internal carotid arteries. Magnetic resonance imaging (MRI) of the brain confirmed an acute right occipital lobe infarction (Figure 3). A transthoracic echocardiogram showed intact left ventricular function with an ejection fraction of 65% and mild diastolic dysfunction. His hospital course was uncomplicated and on postoperative day eight, he was discharged home in hemodynamically stable condition with antiplatelet therapy.

**Figure 4:**
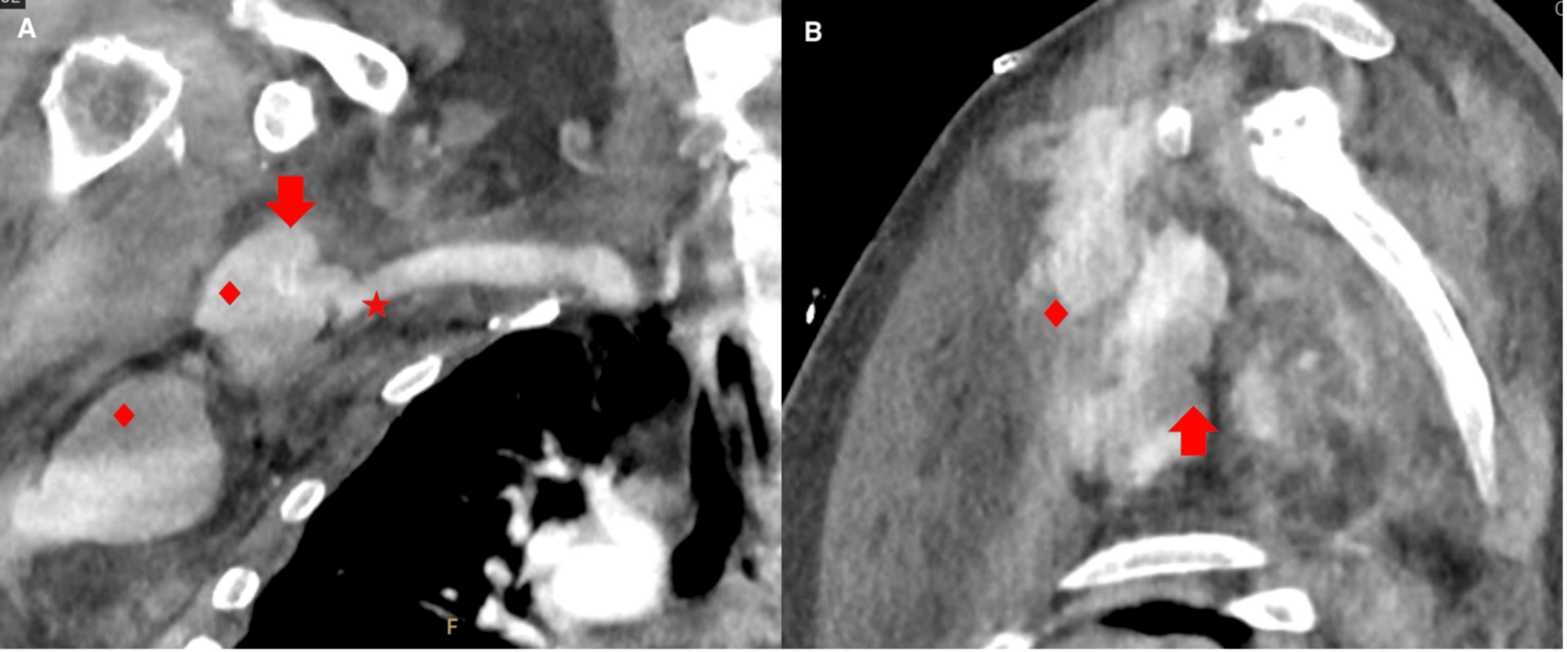
Angiography CT of the right upper extremity The coronal view (A) illustrates the thrombosed bypass stent (arrow), with the pseudoaneurysm emerging off the subclavian artery (star). The surrounding soft tissue shows a collection of fluid containing contrast (diamond) illustrating a developing hematoma in the region. In the sagittal view (B), the thrombosed stent (arrow) can again be visualized, but the pseudoaneurysm is masked by the developing hematoma (diamond).

On postoperative day nine, the patient returned to the hospital due to right upper extremity pain and weakness following a ground-level fall onto an outstretched right hand. A code stroke was initially activated in the emergency room due to new focal deficits. The right upper extremity was flaccid and monoplegic with diminished pulses. Edema or discoloration of the limb was not noted. No further neurologic deficits were appreciated. Neuroimaging, which included MRI and CT, was negative for intracranial hemorrhage, new strokes, or large vessel intracranial arterial occlusions. The patient was not a candidate for thrombolysis or thrombectomy. Right upper extremity imaging was negative for fractures. CT angiography of the right upper extremity showed a thrombosed right axillary-femoral bypass graft with hematoma in the right axillary soft tissue, which was concerning for a pseudoaneurysm compressing the brachial plexus (Figure 4). He underwent emergent hematoma evaluation with right axillary artery stenting. Following the procedure, no further bleeding was noted from that area with a patent axillary artery and flow into the right upper extremity. His pain and motor strength in the right upper extremity improved, and he was eventually discharged home in stable condition.

## Discussion

The development of a focal deficit in the context of recent surgery may raise concern for a cerebrovascular accident. Given this patient’s prior ischemic event, it was appropriate to rule out hemorrhagic transformation based on the patient's presentation upon readmission. With the absence of changes in his prior infarct, stroke mimics needed to be considered. Common stroke mimics include but are not limited to seizures, myasthenia gravis, hypoglycemia, and demyelinating diseases [14,15]. This patient’s unusual presentation of a stroke mimic, characterized by not only weakness but also the atypical and concerning presence of pain, alongside sensory loss, pulselessness, and pallor, underscores the importance of considering traumatic pathologies in an expanded differential once an ischemic stroke has been ruled out.

Management of the patient’s thrombosed artery bypass graft involved surgical stent placement and hematoma evacuation. Stent placement began with establishing brachial artery access. An incision through the skin, subcutaneous tissue, and fascia at the level of the right antecubital fossa was made using a standard sterile technique. The brachial artery was identified, and vessel loops were utilized for control. An IV heparin bolus was administered and access to the brachial artery was achieved with the micropuncture sheath technique with placement of a 7 French sheath. An angiogram was performed to identify the pseudoaneurysm from the axillary artery anastomosis made during AXFBG (Figure 4). A guidewire was advanced beyond the area of the anastomosis with confirmation by an angiogram. A heparin-coated self-expanding stent was deployed into the right axillary artery just distal to the takeoff of the vertebral artery. An angiogram demonstrated brisk blood flow from the axillary artery to the brachial artery with the exclusion of the pseudoaneurysm. The brachial artery access and surgical incision sites were sutured closed. For hematoma evacuation, the patient’s previous incision site over the right neck and axilla was opened. Large amounts of hematoma material were evacuated and a Jackson-Pratt drain was placed. The incision sites were sutured and stapled closed and neurovascular checks every hour were ordered as well as consults with physical, occupational, and speech therapy.

Complications associated with AXFBG include graft kinking, graft thrombosis, seroma, plexus lesions, and arterial steal syndrome [1]. Blunt or penetrative trauma following the AXFBG procedure can create a pseudoaneurysm or hematoma, which can impact the surrounding anatomy including the brachial plexus [12]. Hematomas in this area can mimic stroke symptoms by compressing nerves and causing weakness and pain, much like what was seen in our patient. An efficient method to diagnose and identify the presence of these complications would be through ultrasound and/or computed tomography angiography [12]. Prompt recognition of these early post-surgery issues is vital for clinicians to avert potential harm.

## Conclusions

The AXFBG has been a standard in the treatment of complicated patients with PAD. Although historically successful, complications have been reported that have the potential to complicate the healing process and mimic other conditions, such as stroke, if trauma were to occur to the graft. It is imperative to recognize elements of a patient’s history that put them at risk for a stroke mimic vs an ischemic infarct. Clinicians involved in the post-operative and emergent care of these patients should keep this possibility in mind, as the patient’s limbs and quality of life are at severe risk.
